# Developing an implementation fidelity measure for a family healthy weight program

**DOI:** 10.1186/s12966-025-01755-2

**Published:** 2025-05-06

**Authors:** Caitlin A. Golden, Paul A. Estabrooks, Kate A. Heelan, R. Todd Bartee, Gwenndolyn C. Porter, Emiliane L. Pereira, Bryce M. Abbey, Tzeyu L. Michaud, Jennie L. Hill

**Affiliations:** 1 Population Health Sciences, University of Utah, Salt Lake City, UT 84108 USA; 2 Department of Health and Kinesiology, University of Utah, Salt Lake City, UT 84112 USA; 3 Kinesiology and Sport Sciences Department, University of Nebraska at Kearney, Kearney, NE 68849 USA; 4 Department of Biology, University of Nebraska at Kearney, Kearney, NE 68849 USA; 5 Porter Consulting LLC, Omaha, NE 68101 USA; 6 Department of Health Promotion, University of Nebraska Medical Center, Omaha, NE 68198 USA

**Keywords:** Fidelity, Family healthy weight program, Pediatric obesity, Capacity building, Community engagement

## Abstract

**Background:**

Measuring implementation fidelity is crucial yet proves challenging. While observational methods are considered the gold standard, their practicality in geographically dispersed community settings is often limited by resource constraints. Engaging community members as paid research staff is a potential strategy to develop local capacity to conduct direct observations. This paper reports on the development and preliminary utility of a fidelity measure for a community-based family healthy weight program (FHWP), Building Healthy Families (BHF), and a method to hire and train local community members to conduct direct observation.

**Methods:**

A consensus process guided the development of a comprehensive fidelity measure for direct observation. We piloted and refined the measure using a qualitative iterative approach with observers. Communities delivering BHF were geographically dispersed up to 450 miles resulting in the development of a training protocol to hire and train local community members as direct observers. Inter-rater agreement of ≥ 85% with an expert observer was required for observers to independently assess BHF sessions.

**Results:**

A multidimensional fidelity measure for direct observation was developed specific to the core components of BHF and the session structure and process. The training method successfully prepared community-based observers (*n* = 5) to conduct fidelity assessments with the same quality as the trained research team observers (*n* = 3). Inter-rater agreement ≥ 85% with an expert observer was achieved on all training sessions. The fidelity measure demonstrated strong utility, effectively capturing multiple dimensions of fidelity and provided actionable insights to support consistent and high-quality implementation across community settings.

**Conclusions:**

This study provides a systematic approach to assessing implementation fidelity of a FHWP in micropolitan and surrounding rural areas. Our approach to hiring and training local community members as direct observers enhanced the feasibility of measuring implementation fidelity across multiple geographically dispersed settings and established a model for ongoing assessments.

**Supplementary Information:**

The online version contains supplementary material available at 10.1186/s12966-025-01755-2.

## Background

Comprehensive, multi-component, family healthy weight programs (FHWP) are recommended to reduce child weight status [1]. While FHWPs are shown to be efficacious, there is limited evidence on the generalizability and the extent to which these programs can achieve similar outcomes when implemented in diverse settings [2]. For interventions to be successful when scaled-up and -out, it is important to understand the implementation process and the components necessary to achieve the desired outcomes [3, 4]. Systematically evaluating implementation fidelity is a critical factor to make conclusions about the effectiveness and generalizability of interventions by determining if a failure to produce the desired outcomes was due to low quality implementation or inherent program flaws [5]. The process of measuring fidelity not only provides information on the degree to which an intervention was implemented as intended, but also if any adaptations were made that might influence effectiveness [6]. While definitions and measures vary widely, implementation fidelity typically encompasses five specific dimensions (i.e., adherence to protocol, dose, quality of delivery, participant engagement, and program differentiation; [7]). However, these dimensions are infrequently assessed and reported within studies, limiting their generalizability across different settings and populations [8]. Comprehensive measures that capture all five dimensions of fidelity are recommended to better understand factors that may influence intervention effectiveness [7, 9, 10].

There are various challenges to measuring implementation fidelity within complex behavioral interventions including lack of developed tools, reporting quality, resource constraints for comprehensive monitoring, variability in delivery across different settings and practitioners, and the need to balance fidelity with necessary adaptations for local contexts [7, 8]. In addition, consideration should be given not only to how fidelity will be measured but also who will conduct the assessments. Both direct and indirect measures have been used to measure fidelity within community settings [11–13]. Indirect measures are often used for data collection within community settings through self-reported measures (e.g., surveys or checklist) completed by implementers for their practicality [14]. Self-reported measures are typically less time consuming and resource intensive making them cost-effective, easy to implement, and facilitate longitudinal assessments of fidelity over time [15]. However, self-reported measures are also susceptible to low accuracy and reliability due to implementer bias (e.g., social desirability, recall, response), inconsistencies across implementers, and the potential to mispresent adherence (i.e., under or over report). Additionally, self-reported measures provide limited insight into intervention complexities and may lack generalizability [14–16]. Although more expensive and labor intensive, direct measures, such as observations using trained individuals, provide a more accurate and objective measure of fidelity while providing the potential to give real-time feedback and support quality control that enhances the depth and comprehensiveness of data collected [11, 14, 15, 17].

Despite the importance of measuring fidelity, few studies have reported on the development of fidelity measures and the procedures for sustained monitoring across diverse settings, including community settings [13, 15]. In the context of FHWPs (i.e., obesity prevention and treatment programs), a recent systematic review found that only 18% of pilot/feasibility studies provided a description of how fidelity was assessed and only 14% reported outcomes related to fidelity [18]. Another systematic review conducted by JaKa and colleagues [19] determined dose and the content delivered were often not reported and only one study included all five domains of fidelity. Direct observation and self-reported measures were used equally within these studies, with some using a combination of measures to assess fidelity [13]. The majority of studies reporting on fidelity measures and outcomes are in the clinical or school settings and not family-based [15, 19–22].

While observational methods are more accurate and provide a greater understanding of the implementation process, this method may not be an option due to the expensive and resources needed to conduct direct observations [11, 14, 17]. Furthermore, when interventions occur in community settings and across geographically dispersed areas, using direct observation for measuring fidelity becomes even more complex [6, 15, 23]. Within these contexts, community-engaged research strategies offer a collaborative framework that may address resource constraints while leveraging local knowledge and expertise to ensure accurate and meaningful fidelity assessment [24, 25]. The practice of hiring community-based research assistants is one strategy that has been successful for assessing fidelity by developing community capacity for research [26, 27]. The engagement of community members as paid research staff can also promote high quality process evaluations by leveraging their knowledge and relationships within communities to inform researchers on intervention fit, appropriateness of evaluation measures, and cultural adaptations [28, 29]. Working closely with community-based research assistants allows bi-directional feedback on the implementation process to identify and address challenges at an early stage to mitigate potential adverse effects on the research [29, 30]. Emphasizing community engagement within the evaluation process has the potential to further develop community relationships and collaborations, disseminate scientific knowledge that is informed by local community members, and build capacity to conduct research in micropolitan and surrounding rural areas. The purpose of this paper was to (1) report on the development and preliminary utility of a fidelity measure for a community-based FHWP and (2) describe a method to hire and train direct observers from local communities participating in an ongoing Type III Hybrid Effectiveness-Implementation (type 3) pilot study [31, 32].

## Methods

### Study design

The primary aim of the type 3 study was implementation fidelity with a secondary aim to determine the effectiveness of a FHWP, Building Healthy Families (BHF), when delivered in micropolitan and surrounding rural communities [33, 34]. BHF is an adapted evidence-based FHWP developed and implemented in a micropolitan community through a community–academic partnership to provide a treatment option for families with children who have obesity [35, 36]. Children and their families are eligible to participate in BHF if the child is between 6 and 12 years of age with a BMI ≥ 95 th percentile. BHF consists of 12 weekly face-to-face group sessions, two hours in duration, followed by six refresher sessions (*n* = 18 sessions), 1–2 h in duration, up to one year of cohort implementation to meet the recommended ≥ 26 contact hours with enrolled families. Families engage in nutrition education (Traffic Light Eating Plan), behavior modification focused on key behavior change strategies (e.g., goal setting, self-monitoring, rewards/contingency management, role modeling, and stimulus control/modifying the environment), and physical activity.

Within the type 3 study, a packaged implementation blueprint strategy: BHF Online Training Resources and Program Package (i.e., BHF Resources) was used to support adoption, implementation, and organizational maintenance of BHF. A detailed description of BHF Resources has been published previously [33]. Briefly, BHF Resources includes all the program implementation and training resources necessary to support adoption and implementation through a user friendly online platform. Additionally, the type 3 study included implementing the packaged program in communities with or without participation in a learning collaborative consisting of network weaving, consultee-centered training, goal-setting and feedback, and sustainability action planning. The aim of the study was designed to test the hypothesis that a learning collaborative implementation strategy combined with the BHF Resources would improve program adoption, implementation fidelity, and sustainability when compared with communities with access to the BHF Resources only. Approval for the study was provided by the University of Nebraska at Kearney Institutional Review Board (IRB #121919–1).

The phase of the project described in this paper reports on the development of an implementation fidelity measure for direct observation of BHF in communities (*n* = 7) participating in the type 3 pilot study and a method to hire and train local community members to conduct direct observations. This study did not focus on testing or comparing implementation strategies. The implementation fidelity measure was designed to assess the degree to which community implementation teams (CITs) adhered to the BHF protocol when delivering the program to families using trained individuals to conduct direct observation. We planned for cohort one direct observations to be completed by the research team and any additional cohorts implemented during the type 3 study would be completed by trained community-based observers. Direct observations were scheduled for all 18 sessions in cohort one and any additional cohorts implemented by communities would use a random sample of sessions (*n* = 6) for direct observation.

### Direct observation fidelity measure development

Prior to program implementation, the research team (*n* = 8; including developers of BHF), used a consensus process to develop a comprehensive measure of fidelity for direct observation using Feely et al. [37] field guide to developing a comprehensive fidelity measurement system. To initiate this process, we conducted a review of the literature to identify existing fidelity measures for community-based health promotion interventions. Few studies reported on the development of fidelity measures for direct observation and the majority were within the school setting or not family-based [15, 19–22]. The research team members independently reviewed the findings from the literature review and brought their perspectives to a series of structured discussions. As a result, we found it necessary to create a unique measure of fidelity for a FHWP delivered in community settings guided by Carroll et al. [7] conceptual framework for implementation fidelity and existing measures from the review of the literature [20, 22].

A qualitative iterative approach was used to determine the components of fidelity to be assessed while ensuring alignment with the core components of BHF. Consensus was achieved through group discussion where decisions were made collaboratively to ensure the measure was comprehensive, practical, and grounded in the evidence. We sought to capture the fidelity to the structure and the process of implementing BHF through a multidimensional measure of fidelity. Structure was operationalized as the extent to which CITs delivered the core components of BHF and the process was operationalized as the quality of delivery by CITs [14, 38, 39]. This resulted in a fidelity measure for direct observation with a checklist for activities and objectives completed as well as a 4-point scale specific to each BHF session (1 = topic was not covered, 2 = topic was covered, 3 = topic was covered with good examples 4 = topic was covered with good examples and participants were engaged; Additional File 1).

### Direct observation fidelity measure utility testing

Community implementation of BHF was staggered and began in March of 2021. We piloted our fidelity measure for direct observation in two communities. During weekly research team meetings a qualitative iterative process was used to discuss the implementation process and the utility of the fidelity measure after each observed session. The research team observers encountered difficulty in effectively capturing specific fidelity components and deviations within the session structure and quality of delivery while assessing BHF sessions. The research team reached consensus to refine the measure after discussions with observers and re-reviewing the literature of existing fidelity measures [20] to allow for a comprehensive assessment of five components of fidelity and distinct items addressing the session process and structure [7]. This process resulted in the development of a refined fidelity measure for direct observation with a 3-point scale (0 = did not cover, 1 = partially covered, 2 = fully covered) that captured the core BHF components and session specific objectives. Dichotomized (yes/no) items specific to each session were also included for activities completed per the protocol (i.e., the provided lesson plan) and if adaptations occurred during the observed session. If adaptations occurred, a space was provided for observers to provide specific details on the nature of the adaptation. Additional quality measures were incorporated to the refined fidelity measure for direct observation by adding the lesson plan outline with suggested timing (i.e., duration of each activity) and a scoring rubric (i.e., rater dictionary) for the direct observers to reference during sessions [7, 20] (Additional File 2).

### Direct observation training protocol

After completing a request for applications process to identify and select communities ready to adopt and implement BHF [40], the proximity of the research institute to communities participating in the type 3 study spanned a distance up to 450 miles and was not feasible for the research team to complete all direct observations or an efficient use of resources. To address this, an expert observer (PE) on the research team (i.e., a senior researcher with expertise in FHWP evaluation and dissemination and implementation science) recorded the first five BHF sessions during direct observations to allow for future training opportunities. Additionally, two research team members (CG, GP) with prior experience conducting direct observations and implementing BHF co-observed the first two sessions virtually with the expert observer and independently completed fidelity assessments. Based on correspondence of ratings (> 85% agreement) between the research team members and the expert observer, the research team members were able to assess community sessions independently and were now considered expert observers. Subsequently, the expert observers (*n* = 3) collaboratively developed a training protocol for onboarding new direct observers.

The training protocol provided an overview of the (1) intent of each item on the fidelity measure for direct observation, (2) level of quality of implementation, and (3) direct observation and data collection protocol when in communities. Local direct observers were recruited through job advertisements in communities delivering BHF via the local paper, community colleges, and Indeed. Ideal applicants were described as having a high school degree, plus 2 years of college or technical training and 2 years of research or practice experience in health, health promotion or education, or any other related field. Once the onboarding paperwork was complete, community-based observers met with an expert observer (CG, GP) over a video conferencing platform to initiate training. Prior to the meeting with the expert observer, community-based observers were given access to the BHF Resources and asked to complete the program overview training module to learn about the program and pass the accompanying knowledge check to assess their understanding of the program. Upon successfully completing the BHF program overview training, community-based observers met with an expert observer to review BHF, implementation fidelity, the fidelity measure and scoring criteria, and the procedures for observing in a community, data collection, and reporting.

**Fig. 1:**
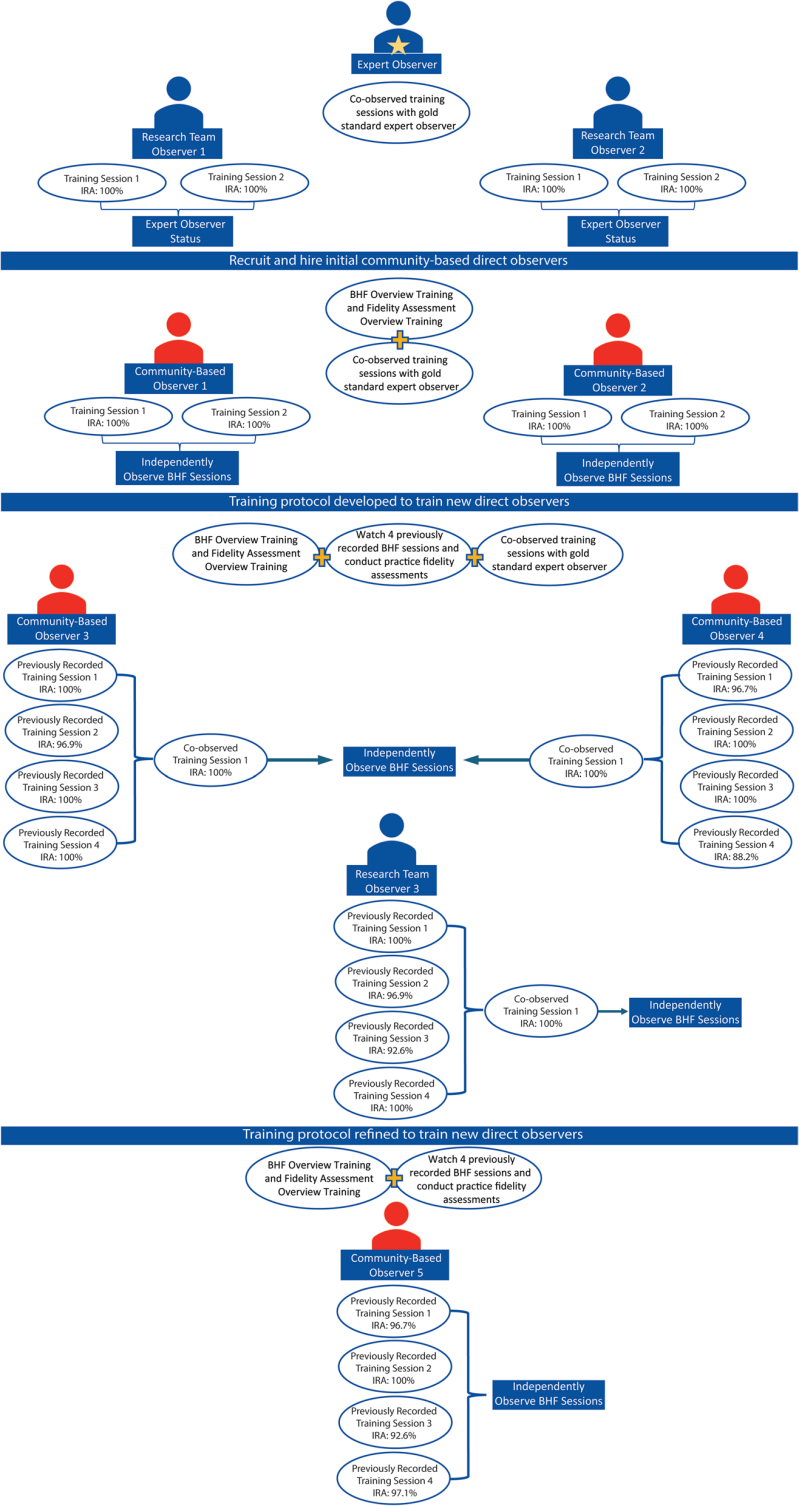
Direct observer training protocol and inter-rater agreement

The COVID-19 pandemic caused implementation of BHF to be staggered. To meet the needs of the type 3 study and timeline of CIT implementation, two initial community-based observers were trained. As communities began implementing BHF, the research team’s capacity to observe all communities in a geographically dispersed area required the training protocol to be adapted as more direct observers were needed. Figure 1 provides an overview of the direct observer training protocol as the pilot study progressed. Observers were rated within a margin of error (± 1 on the same end of the rating scale) relative to the expert observer and were required to achieve > 85% inter-rater agreement with the expert observer to independently observe BHF sessions. If an observer did not reach > 85% agreement with the expert observer, the protocol required observers to attend additional sessions with an expert observer until > 85% agreement was achieved.

The two initial community-based observers were required to complete the BHF overview training and fidelity assessment overview training with the expert observer before attending two consecutive sessions with the expert observer. As more CITs began implementing BHF, additional training requirements were incorporated for onboarding new direct observers in addition to the BHF overview training and fidelity assessment overview training. The change to the training protocol required observers to review the previously recorded BHF sessions (*n* = 4) and complete practice fidelity assessments for each session before co-observing a session with an expert observer.

The previously recorded BHF sessions were shared with observers through a secured data sharing platform during the initial training. The observers were instructed to complete the session specific trainings with knowledge checks prior to watching the corresponding recorded BHF session and to complete the practice fidelity assessment in real-time. The practice fidelity assessments were reviewed and compared to the expert observer and a follow up meeting was scheduled to review scores and address any questions posed by observers. Observers (*n* = 3) then attended a session with an expert observer, independently completed a fidelity assessment, and compared scores following the session. The training protocol was refined and finalized to onboard any additional observers by removing the requirement to co-observe a session with an expert observer if > 85% inter-rater agreement was reached across all previously recorded BHF practice sessions. If an observer did not reach > 85% agreement across the previously recorded sessions the protocol required observers to attend a session with an expert observer until > 85% agreement was achieved.

### Direct observation process

The BHF CIT program coordinators were informed that a direct observer would be present for each of the program sessions and was explained as a component of the type 3 study to assess each session for completeness and opportunities for program improvement. Prior to observing each session, the observers were required to review the corresponding online BHF training, session materials, and the fidelity assessment at least one day in advance. Observers were asked to arrive 15 min prior to the session start time, introduce themselves to the BHF coordinators if it was their first observation, and find a quiet, inconspicuous, location to complete the fidelity assessment. During the session, observers completed the fidelity assessment in real time and were instructed to take detailed notes on the implementation process and any adaptations made by community implementation teams. After the session was completed, direct observers thanked the coordinators for allowing them to observe the session, inquired the status of coordinator knowledge checks, and reminded the program coordinator to complete the self-report fidelity checklist for the session. Each session was reported back to the research team by the direct observers during weekly, 60-min lab meetings. Direct observers were compensated for their time and mileage to conduct direct observations and their time attending weekly lab meetings.

## Results

**Fig. 2:**
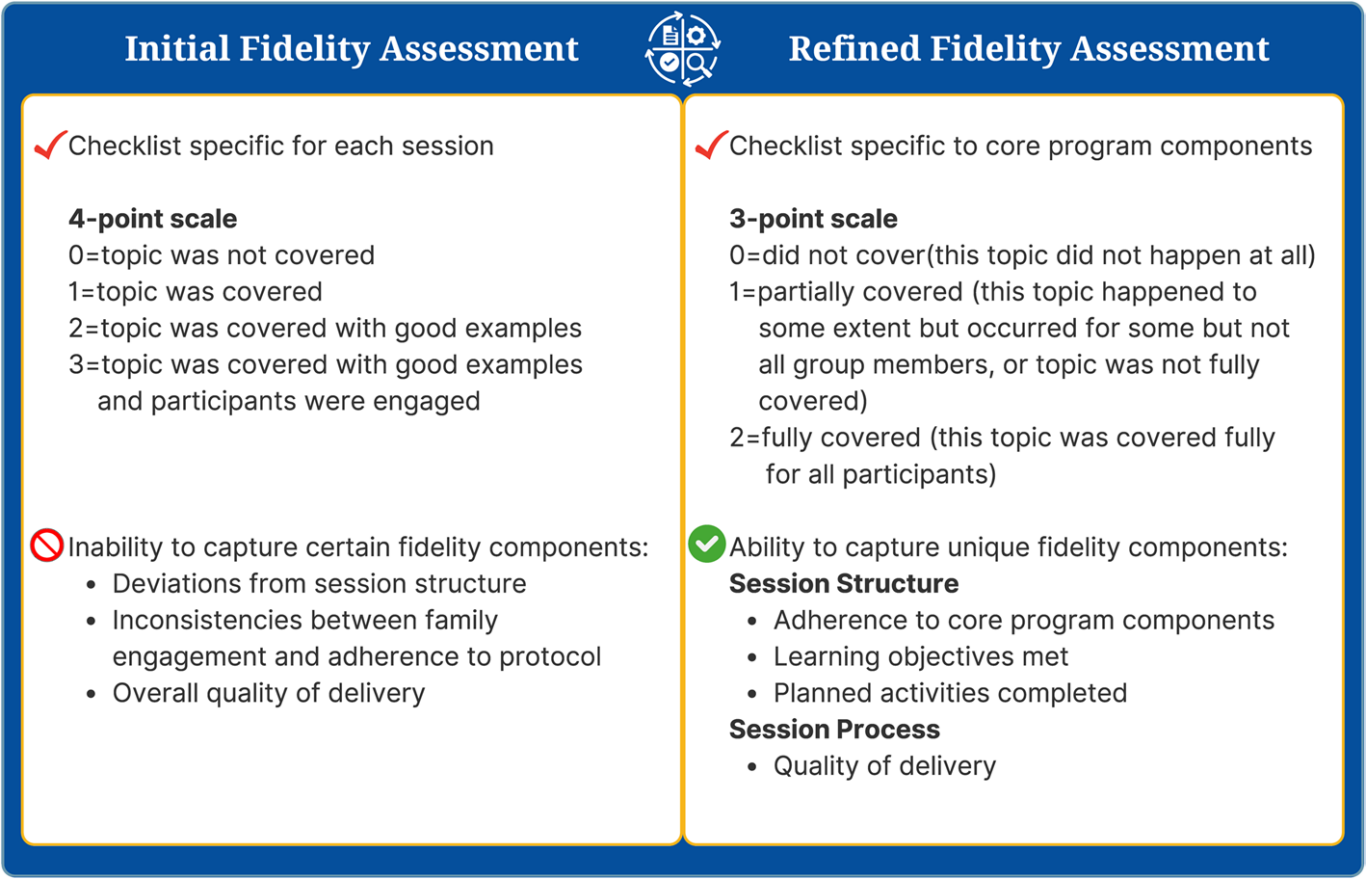
Comparison of the initial and refined fidelity measure for direct observation

The consensus process identified three core components of the BHF program to be measured across sessions; 1) Traffic Light Eating Plan curriculum (nutrition and lifestyle modification strategies), 2) self-regulation (goal setting and strategies for overcoming barriers), and 3) physical activity (child only and child with family components). The refined fidelity measure specific to BHF core components with the 3-point scale demonstrated enhanced ease of use among direct observers and a more comprehensive measure of fidelity. As a result, a multidimensional measure was created that captured the fidelity to the structure (e.g., adherence, dose) and the process (e.g., participant responsiveness/engagement, quality of delivery, program differentiation) of implementing BHF. Figure 2 provides a comparison of the initial and refined fidelity measure for direct observation. *Adherence* was defined as the extent to which the program content (curriculum), frequency, and duration was implemented as directed by protocol. The *dose* was defined as the total number of sessions delivered by the length of sessions (contact hours). *Participant Responsiveness (engagement)* was defined as the extent to which the participants were engaged with the program activities, the other participants (families), and the CIT coordinators delivering the sessions. *Quality of Delivery* was defined as the manner in which the CIT coordinators delivered BHF (e.g., preparedness, friendly and empathetic, class management). *Program Differentiation* was defined as the presence or absence of the core BHF components with adaptations made by community implementation teams being documented qualitatively.

**Fig. 3:**
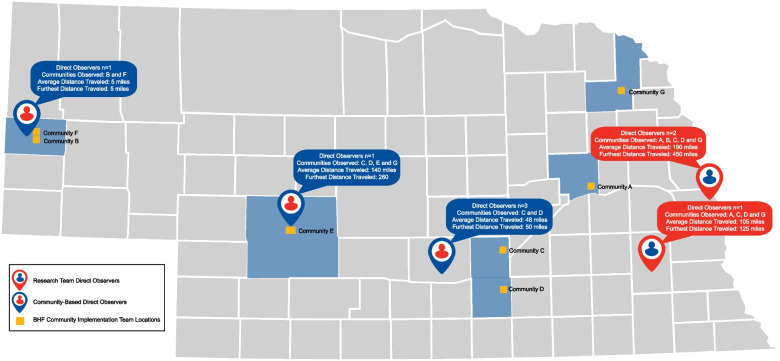
Geographical distribution of direct observers and assessed communities

Five community-based observers were recruited and hired in addition to the three observers from the researcher team. Figure 3 displays the geographical distribution of community implementation team locations and direct observer departure points. Community-based observers were located throughout western (*n* = 1), west-central (*n* = 1), south-central (*n* = 3), Nebraska and research team observers were located in southeastern (*n* = 1), and eastern (*n* = 2) Nebraska. The furthest distance traveled to a community delivery site by a community-based observer was 260 miles (one-way). Expert observers from the research team traveled up to 450 miles (one-way) before implementation of the direct observer training, reducing the furthest distance traveled to a community delivery site to 160 miles (one-way). Community-based observers attended approximately 60 virtual dissemination and implementation research team meetings. Overall, 135 direct observations (52 research team observations and 83 community-based observations) occurred between March of 2021 and April of 2024 (Table 1).

**Table 1:** Frequency of direct observations by research team observers and community-based observers

*Cohort*	Community							
	A	B	C	D	E	F	G	Total
1	*n* = 12 RTO *n* = 12 CBO *n* = 0	*n* = 18 RTO *n* = 7 CBO *n* = 11	*n* = 9 RTO *n* = 2 CBO *n* = 7	*n* = 14 RTO *n* = 8 CBO *n* = 6	*n* = 18 RTO *n* = 0 CBO *n* = 18	*n* = 17 RTO *n* = 0 CBO *n* = 17	*n* = 14 RTO *n* = 13 CBO *n* = 1	*n* = 102 RTO *n* = 42 CBO *n* = 60
2	N/A^a^	*n* = 4 RTO *n* = 0 CBO *n* = 4	*n* = 3 RTO *n* = 0 CBO *n* = 3	*n* = 4 RTO *n* = 3 CBO *n* = 1	*n* = 6 RTO *n* = 0 CBO *n* = 6	*n* = 6 RTO *n* = 0 CBO *n* = 6	*n* = 4 RTO *n* = 4 CBO *n* = 0	*n* = 27 RTO *n* = 7 CBO *n* = 20
3	N/A^a^	N/A^a^	N/A^a^	*n* = 4 RTO *n* = 1 CBO *n* = 3	N/A^a^	N/A^a^	*n* = 2 RTO *n* = 2 CBO *n* = 0	*n* = 6 RTO *n* = 3 CBO *n* = 3
*Total*	*n* = 12 RTO *n* = 12 CBO *n* = 0	*n* = 22 RTO *n* = 7 CBO *n* = 15	*n* = 12 RTO *n* = 2 CBO *n* = 10	*n* = 22 RTO *n* = 12 CBO *n* = 10	*n* = 24 RTO *n* = 0 CBO *n* = 24	*n* = 23 RTO *n* = 0 CBO *n* = 23	*n* = 20 RTO *n* = 19 CBO *n* = 1	*n* = 135 RTO *n* = 52 CBO *n* = 83

*Abbreviations*: *RTO* Research Team Observations, *CBO* Community-Based Observations

^a^N/A, community did not implement cohort

The trained research team members reached 100% agreement within two co-observed sessions with the gold standard expert observer. The two initially trained community-based observers also achieved 100% agreement within two co-observed sessions with an expert observer. Inter-rater agreement > 85% was achieved for all community-based observers throughout the four previously recorded BHF training sessions and 100% agreement during the co-observed session with an expert observer (Fig. 1).

## Discussion

This study contributes to the literature by sharing a process to develop a measure of fidelity for direct observation within the context of a community-based FHWP and a method to hire and train local community members to conduct direct observations. In general we found that a focus on core intervention components and the use of simple response scales with a rater dictionary was more acceptable for direct observers and our training prepared community-based observers to conduct direct observations similarly to those who were trained members of our research team. Our training and close collaboration with community-based observers resulted in a direct observation process that can potentially be scaled up to assess fidelity in rural areas of the United States.

Our approach to develop a comprehensive measure of fidelity for direct observation resulted in a multidimensional assessment that captured the fidelity to the structure (e.g., adherence, dose) and the process (e.g., participant responsiveness, quality of delivery, program differentiation) of implementing a FHWP. We extensively piloted the fidelity measure for direct observation and through a qualitative iterative process with observers, refined the assessment into a more intuitive measure that was specific to the core components of BHF and able to detect more variability within the dimensions of fidelity. The fidelity measure demonstrated strong utility and provided actionable insights to support consistent and high-quality implementation across community settings.

The direct observation training effectively prepared community-based observers to assess BHF sessions independently. Similar to other studies who have trained individuals outside of the core research team to conduct direct observations [22, 41], our community-based observers reached > 85% agreement with the expert observer across all previously recorded training sessions and within the co-observed in-person sessions. Suggesting our direct observer training was sufficient to achieve high quality and reliable observations and the expert observer (i.e., the gold standard observer) co-observed sessions may not be necessary when training future community-based observers. Although this process to hire and train local community members to conduct direct observations required a significant investment in time and resources to execute, process evaluations are a critical component of program evaluation to understand the relationship between the intervention and the context and how outcomes are achieved [42]. Training and deploying both research team members and local community members for direct observation allowed our team to utilize resources more efficiently and responsibly while collecting high quality data on the implementation process of a FHWP delivered in multiple micropolitan communities within a type 3 study.

Although challenging, consistent, high quality measurement of fidelity is essential to determine which components of an intervention must be maintained to achieve outcomes and can enhance the replicability of an intervention [14]. Implementation fidelity can significantly influence the effectiveness of an intervention when implemented in diverse settings due to differences in the delivery setting and implementation personnel, intervention program features, participant characteristics, and community resources [43, 44]. Implementation should involve a degree of tailoring for the local context while maintaining the intervention core components [6] though adaptations are typically avoided due to concerns over reductions in efficacy, which can lead to program drift and decreased effectiveness [45], underscoring the importance of long-term fidelity monitoring to ensure quality, effectiveness, and sustainability of programs implemented across diverse settings [45, 46]. Conducting direct observations throughout our type 3 pilot study not only allowed for adaptations to be documented but also for implementation issues to be identified and addressed in real time to prevent program drift. Additionally, direct observations provided an opportunity for the research team to identify common skill building opportunities for CIT coordinators that targeted improvements in implementation quality and outcomes through the learning collaborative condition of our study.

An unforeseen benefit to this process was the facilitation of bi-directional feedback on the implementation process and relationship building between the research team and community-based observers with the local community implementation teams. The collaborative involvement of community-based observers with the research team during weekly meetings fostered community engagement and provided key insights into implementation of a FHWP which otherwise would have been unknown to researchers [47]. Incorporating a community-engaged approach during the evaluation process allowed researchers to directly incorporate community partners’ expertise and lived experience into a type 3 study, improving both the implementation process and findings [29, 30]. Fostering successful community-academic research partnerships require capacity building efforts to facilitate adoption, implementation, and sustainability of evidence-based interventions. The process we outlined to hire and train local research assistants as community-based direct observers demonstrated one way to build capacity to implement a FHWP through engaging community members within the research process. There were three critical steps to maintaining high reliability in measuring fidelity across direct observers and included the 1) comprehensive training process for direct observers 2) implementation of a detailed rater dictionary for the fidelity assessment and 3) weekly meetings with the dissemination and implementation team [48].

Our study provides a systematic approach to assessing implementation fidelity of a FHWP in micropolitan and surrounding rural areas using a multidimensional measure. Understanding the fidelity of specific components within a FHWP is essential to identify which components of an intervention contribute to outcomes and provides guidance to translate interventions into diverse settings. The value of having trained community-based observers present in communities and as participants within the research team meetings has surmounted expectations through new knowledge production and dissemination as well as meaningful engagement within communities. Our team has incorporated this process into future funding applications and community-based observers have continued their engagement as consultants in scale up efforts and as advocates within their communities.

## Conclusions

The successful development of an implementation fidelity measure and a method to hire and train local community members as direct observers is critical to ensure consistent and effective delivery of a FHWP in community settings. Our approach to using local community members as direct observers enhanced the feasibility of measuring implementation fidelity across multiple geographically dispersed settings and established a model for ongoing assessments.

## Supplementary Information

Additional file 1.

Additional file 2.

## Data Availability

The datasets used and/or analyzed during the current study are available from the corresponding author on reasonable request.

## Abbreviations

BHF: Building Healthy Families
CBO: Community-Based Observers
FHWP: Family Health Weight Program
IRA: Inter-Rater Agreement
RTO: Research Team Observers
TLEP: Traffic Light Eating Plan
