# Supplementary material for: Developing an implementation fidelity measure for a family healthy weight program
Source: Int J Behav Nutr Phys Act. 2025 May 6;22:53. doi: 10.1186/s12966-025-01755-2 (PMC12057106; doi:10.1186/s12966-025-01755-2)
Supplement: Supplementary file 1 — Additional file 1. [file 12966_2025_1755_MOESM1_ESM.docx]

**Session:** 3- Creating Balance

**Meeting Time:**

**Session Duration:**

**Date:**

**Start Time:**

**End Time:**

**Community:**

**Number of Families:**

**Number of Participants:**

**Observer:**

| **Activities** | **Session Timeline** |
| --- | --- |
| 1. Weigh in and Welcome 2. Count the Reds Game- review red foods 3. Energy Balance Presentation and activity 4. Kids go to activity: Kin-Ball 5. Discussion with parents on Energy Balance 6. Family physical activity: circuit training and home exercises 7. Questions | 15 minutes weigh-in and welcome  20 minutes Count the Reds Game  15 minutes Energy Balance Presentation  25 minutes kid physical activity and discuss energy balance with parents  30 minutes Family physical activity  15 minutes questions and wrap-up |

**Session objectives Completed:** ____ Total Score (count number of boxes checked) out of ____ = ____%

Participants will obtain knowledge about energy balance.

Participants will learn about positive and negative energy balance.

Participants will become aware of the importance in identifying barriers and planning to overcome them.

**Activities Completed:** ____ Total Score (count number of boxes checked) out of ____ = ____%

Weigh-in station set up

Handout printouts prior to start

Handed out Habit Books

TLEP Red Foods overview

Count the Reds Game

Energy Balance Activity

Kid’s physical activity

Parent and child physical activity

**Session Facilitation:** Please rate the following activities:

| Weigh in families | 1- Families were not weighed in.  2- Some families were weighed in.  3- Most of the families were weighed in.  4- All of the families were weighed in. |
| --- | --- |
| **Traffic Light Eating Plan** | |
| Count the red foods | 1- Coordinators did not instruct on how to count red foods.  2- Coordinators provided a description of how to count red foods.  3- Coordinators provided a description of red foods and gave examples to families to identify red foods.  4- Coordinators provided a description of red foods, gave examples, and really kept the families participating. |
| Energy Balance | 1- Coordinators did not instruct on energy balance.  2- Coordinators provided an overview of energy balance.  3- Coordinators provided an overview of energy balance and gave families their snacks.  4- Coordinators provided an overview of energy balance, gave families their snacks, and really kept the families participating. |
| Self-Regulation | 1-Coordinators did not review the importance of identifying barriers and planning to overcome them.  2- Coordinators reviewed the importance of identifying barriers and planning to overcome them.  3- Coordinators provided an overview and set family goals for the week.  4. Coordinators provided an overview, set goals for the week, and really kept the families participating. |
| Physical Activity | 1- Physical activity session was not completed.  2- The parents/guardians did not participate during physical activity.  3- Most of the family members were active, having fun, and engaged during physical activity.  4- All of the family members were active, having fun, and engaged during physical activity. |
| Overall Family engagement | 1- Families were not engaged with the coordinators and other participants during the discussion and activities.  2- Families were engaged with the coordinators and other participants **in some** of the discussion and activities.  3- Families were engaged with the coordinators and other participants **in most** of the discussion and activities but not all of them.  4- Families were engaged with the coordinators and other participants **in all of** the discussion and activities. |
| Quality of Delivery | 1- Materials were not ready for the session.  2- Materials were ready for the session, but family questions weren’t always answered.  3- Materials were ready for the session and family questions were answered.  4- All materials were ready for the session, all family questions were answered, and the coordinator really engaged participants. |
| Session Schedule | 1- The scheduled time for activities was not followed.  2- Some activities stayed on schedule.  3- Most activities stayed on schedule.  4- All activities stayed on schedule. |

***Use the back of this form for any comments about the session (were any activities changed):**
