# Supplementary material for: Developing an implementation fidelity measure for a family healthy weight program
Source: Int J Behav Nutr Phys Act. 2025 May 6;22:53. doi: 10.1186/s12966-025-01755-2 (PMC12057106; doi:10.1186/s12966-025-01755-2)
Supplement: Supplementary file 2 — Additional file 2. [file 12966_2025_1755_MOESM2_ESM.docx]

**Session:** 3- Creating Balance

**Meeting Time:**

**Session Duration:**

**Date:**

**Start Time:**

**End Time:**

**Community:**

**Number of Families:**

**Number of Participants:**

**Observer:**

| **Activities** | **Session Timeline** | **Actual Time Spent on Activity** |
| --- | --- | --- |
| First Family Arrival Time:  Last Family Arrival Time:   1. Weigh in and Welcome 2. Count the Reds Game- review red foods 3. Energy Balance Presentation and activity 4. Kids go to activity: Kin-Ball 5. Discussion with parents on Energy Balance 6. Family physical activity: circuit training and home exercises 7. Questions | 15 minutes weigh-in and welcome  20 minutes Count the Reds Game  15 minutes Energy Balance Presentation  25 minutes kid physical activity and discuss energy balance with parents  30 minutes Family physical activity  15 minutes questions and wrap-up | Minutes  Minutes  Minutes  Minutes  Minutes  Minutes |

**Please rate the session objectives:**

| 0 | 1 | 2 |
| --- | --- | --- |
| Did not cover=this objective did not happen at all. | Partially covered=this objective happened to some extent, but the facilitator did not fully cover the session material to meet this objective. | Fully covered=the objective was met fully for the session. |

| **Objective** | **Score** |
| --- | --- |
| Participants will obtain knowledge about energy balance. |  |
| Participants will learn about positive and negative energy balance. |  |
| Participants will become aware of the importance in identifying barriers and planning to overcome them. |  |

**Activities Completed:** ____ Total Score (count number of boxes checked) out of ____ = ____%

Weigh-in station set up

Handout printouts prior to start

Handed out Habit Books

TLEP Red Foods overview

Count the Reds Game

Energy Balance Activity

Kid’s physical activity

Parent and child physical activity

**Please rate the session structure:**

| 0 | 1 | 2 |
| --- | --- | --- |
| Did not cover=this topic did not happen at all. | Partially covered=this topic happened to some extent but occurred  for some but not all group members, all  of the time or was not fully covered (e.g., coordinator facilitated discussion but only among certain members of the group, coordinator did not cover all of the curriculum for the session) | Fully covered=the topic was met fully for all participants. |

| **Component-** Traffic Light Eating Plan Curriculum | **Score** |
| --- | --- |
| Coordinators delivered the nutrition curriculum provided. |  |
| Coordinators delivered the lifestyle modification curriculum provided. |  |
| Coordinators provided examples to families during the nutrition lesson. |  |
| Coordinators provided examples to families during the lifestyle modification lesson. |  |

| **Component-** Self Regulation | **Score** |
| --- | --- |
| Families were weighed in. |  |
| Coordinator collected habit books and distributed new ones to families. |  |
| Coordinator elicits discussion about success/failures since last session. |  |
| Coordinator initiated discussion about problem-solving approach to address barriers. |  |
| Coordinator prompts review of goal attainment. |  |
| Participants set goals for next session. |  |

| **Component-** Physical Activity | **Score** |
| --- | --- |
| Children were active during family physical activity. |  |
| Parents were active during family physical activity. |  |
| Children had fun during family physical activity. |  |
| Parents had fun during family physical activity. |  |

**Please rate the session process:**

| 0 | 1 | 2 |
| --- | --- | --- |
| Did not demonstrate=this process was not demonstrated at all. | Inconsistently demonstrated=this process happened to some extent but occurred  for some but not all activities. | Consistently demonstrated= this process was demonstrated consistently and appropriately through the entire session. |

| **Component-** Family Engagement | **Score** |
| --- | --- |
| Parents were engaged with the coordinators during the educational session. |  |
| Parents were engaged with each other during the educational session. |  |
| Children were engaged in the educational session. |  |
| Parents were engaged during physical activity. |  |
| Children were engaged during physical activity. |  |

| **Component-** Quality of Delivery | **Score** |
| --- | --- |
| The coordinators delivered the planned session for the night. |  |
| Materials were ready for the session |  |
| Coordinators delivered the material in a friendly and empathetic way that facilitated discussion. |  |
| Coordinators encouraged participants to seek feedback about the session content. |  |
| Questions posed by families were answered. |  |
| Coordinators really engaged participants. |  |
| The coordinators controlled/minimized distractions and side conversations from participants and the agenda was not disrupted. |  |
| The activities stayed on schedule with the lesson plan. |  |

| **Component-** Adaptations | **Yes/No** |
| --- | --- |
| Did Facilitators start weighing families at the start time? |  |
| Did Facilitators make any positive adaptations? (Implemented an activity or changed the lesson plan that made a positive impact on participants) |  |

***If yes, what were the adaptations made?**

***Please provide any comments about the session below (were any activities changed):**

**Coordinator Check-in**

**Coordinator 1:**

**Training:** Yes/No

**Knowledge Check:** Yes/No

**Questions:**

**Coordinator 2:**

**Training:** Yes/No

**Knowledge Check:** Yes/No

**Questions:**

**Coordinator 3:**

**Training:** Yes/No

**Knowledge Check:** Yes/No

**Questions:**

**Coordinator 4:**

**Training:** Yes/No

**Knowledge Check:** Yes/No

**Questions:**
